# Immune Checkpoint Inhibitor-Associated Pneumonitis in Non-Small Cell Lung Cancer: Current Understanding in Characteristics, Diagnosis, and Management

**DOI:** 10.3389/fimmu.2021.663986

**Published:** 2021-05-28

**Authors:** Qin Zhang, Liansha Tang, Yuwen Zhou, Wenbo He, Weimin Li

**Affiliations:** ^1^ Department of Respiratory and Critical Care Medicine, West China Hospital, Sichuan University, Chengdu, China; ^2^ Department of Postgraduate Student, West China Hospital, Sichuan University, Chengdu, China; ^3^ Department of Biotherapy, Cancer Center, West China Hospital, Sichuan University, Chengdu, China; ^4^ Department of Neurosurgery, West China Hospital, Sichuan University, Chengdu, China

**Keywords:** immune checkpoint inhibitor, pneumonitis, non-small-cell lung cancer, diagnosis, management

## Abstract

Immunotherapy that includes programmed cell death-1 (PD-1), programmed cell death- ligand 1 (PD-L1) and cytotoxic T lymphocyte antigen 4 (CTLA-4) inhibitors has revolutionized the therapeutic strategy in multiple malignancies. Although it has achieved significant breakthrough in advanced non-small cell lung cancer patients, immune-related adverse events (irAEs) including checkpoint inhibitor pneumonitis (CIP), are widely reported. As the particularly worrisome and potentially lethal form of irAEs, CIP should be attached more importance. Especially in non-small cell lung cancer (NSCLC) patients, the features of CIP may be more complicated on account of the overlapping respiratory signs compromised by primary tumor following immunotherapy. Herein, we included the previous relevant reports and comprehensively summarized the characteristics, diagnosis, and management of CIP. We also discussed the future direction of optimal steroid therapeutic schedule for patients with CIP in NSCLC based on the current evidence.

## Highlights

- Immune checkpoint inhibitor-associated pneumonitis in non-small cell lung cancer presents complicated clinical and radiological manifestations.
- The management of corticosteroids combined with immunosuppressive drugs is deemed to be effective for immune checkpoint inhibitor-associated pneumonitis.
- Patients with immune checkpoint inhibitor-associated pneumonitis tend to suffer from a poor prognosis.

## Introduction

Lung cancer has the greatest death rate, at 25%, of all types of cancer, with an estimated 135,720 deaths in the United States in 2020 (1). Non–small cell lung cancer (NSCLC) is the most common lung cancer subtype, and it comprises two major histological types: squamous cell carcinoma (SCC) and adenocarcinoma (AC) (2). Nearly 70% of patients with NSCLC are initially diagnosed at a locally advanced stage and suffer from a poor prognosis (2). The 5-year survival rate is less than 3% for patients with advanced NSCLC (3). Historically, the standard management recommended for patients with NSCLC who present with advanced-stage disease was chemotherapy regimens combined with radiotherapy (RT). However, the treatment provided generally modest responses, with an overall survival (OS) of approximately 12 to 18 months and a median progression-free survival (PFS) of just 4 to 8 months (4, 5).

Recently, immunotherapy that includes programmed cell death-1 (PD-1), programmed cell death ligand 1 (PD-L1), and cytotoxic T-lymphocyte antigen 4 (CTLA-4) inhibitors, which enhance anti-tumor activity, has revolutionized the therapeutic strategy for multiple malignancies (6). PD-1, a type I transmembrane protein, exists inherently on activated T cells, B cells, natural killer cells, macrophages, dendritic cells, and monocytes. PD-L1 is highly expressed on both cancer cells and antigen-presenting cells (7). The interaction of these two molecules could promote self-tolerance and attenuate autoimmunity through T-cell exhaustion and reduced cytokine production (8). CTLA-4, a critical surface protein receptor and co-inhibitor, is typically located in stimulated CD4+/CD8+ T cells to dampen T-cell activity by binding CD80/CD86/CD28. Using the inhibitory mechanism checkpoint pathways or molecules, immune checkpoint inhibitors (ICIs) can tilt the immune equilibrium toward the beneficial promotion of tumor killing and the boosting of an immune attack (6, 9).

In advanced NSCLC, an increasing body of clinical studies suggests that the application of ICIs could achieve significant breakthroughs in PFS and OS (10–13). Therefore, the US Food and Drug Administration has rapidly incorporated ICIs into first-line therapies for advanced NSCLC (14). In the PACIFIC regimen, durvalumab (a PD-L1 inhibitor) has become the new standard of care after platinum-based chemoradiotherapy for unresectable stage III NSCLC in the United States, Europe, and Japan (15).

However, along with the killed tumor cells, virtually every organ system could be affected by ICIs (5). Immune-related adverse events (irAEs), such as cutaneous lesions, myocarditis, hepatitis, colitis, endocrinopathies, inflammatory arthritis, and pneumonitis, are widely reported (15, 16). The incidence of irAEs might be higher with combination ICI use, specific cancer types, and non-trial conditions (17, 18). Among all reported irAEs, checkpoint inhibitor pneumonitis (CIP) is particularly worrisome and potentially lethal (18–21). CIP may occur more often and have a faster onset in NSCLC than in other types of cancer (22). Since before ICI therapies, pulmonary function has been compromised by tumor location and size in patients with NSCLC. In addition, pre-existing lung comorbidities, such as chronic inflammatory respiratory diseases, interstitial fibrosis lung diseases, and radiation-induced pneumonitis (RIP), may cloud diagnostic accuracy because of the overlapping respiratory symptoms and signs (5, 6, 9, 14, 23). As a result, recognizing the unique clinical and imaging patterns of CIP is essential to facilitate expeditious diagnosis and optimized management principles.

Although previous studies have elucidated the incidence, potential mechanisms, diagnosis, risk factors, and management of CIP, they focused on variable focuses that were not comprehensive and deep enough (5, 6, 9, 14, 23, 24). This review offers a summary of cases or case series concerning CIP in NSCLC, and it aims to identify the characteristics of typical patients who develop CIP. We also comprehensively summarize the current knowledge and relevant studies of ICI-associated pneumonitis, and we discuss the future direction of evidence-based therapeutic schedules for patients with CIP in NSCLC.

## Incidence and Onset of CIP

The definition of CIP is the occurrence of respiratory symptoms/signs related to a new emerging infiltration viewed on a chest X-ray but excluding new infections tested by sputum and/or bronchoalveolar lavage (BAL) (5). In different tumor types, the overall incidence of CIP varied from 3% to 5% for all grades and ranged from 0.8% to 1.0% for grade ≥ 3 CIP (5, 14, 25, 26). The overall fatality rate of CIP was 10% to 17%. In NSCLC, the incidence of CIP mainly originated from clinical trial and real-world data. In clinical trial data (10, 27–44), the incidence of CIP for all grades was approximately 2% to 38%, and incidence for grade ≥ 3 CIP was approximately 0.6% to 2.7%. In real-world data, the incidence of CIP in patients with NSCLC was 4.8% to 39.3% (18, 24, 27, 28, 45–52). The discrepancy between data from these two sources might be partly attributed to the increasing awareness of CIP in the medical community, which contributed to more frequent clinical detection and less stringent inclusion criteria for real-world studies compared with randomized trials.

**Figure 1:**
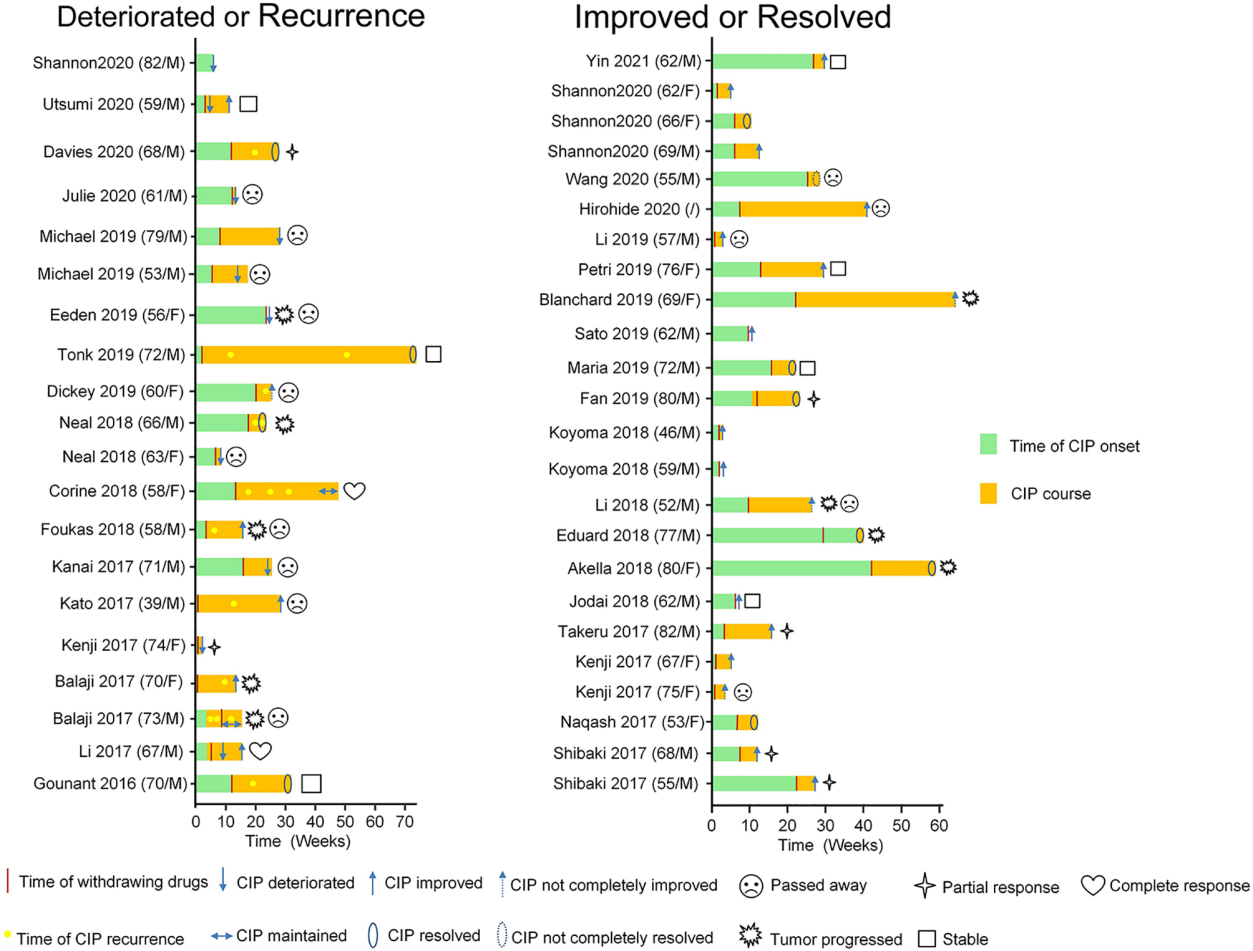
Summary of checkpoint inhibitor pneumonitis patients with NSCLC, including deteriorated or recurrence (n = 20) and improved or resolved (n = 24) patients with more details in **Table 1**.

The median time to the onset of CIP was typically approximately 2.8 months, and the overall range spanned from 9 days to 19.2 months (18, 20, 53, 54). We included 44 occurrences of CIP in patients with NSCLC (**Figure 1**; **Table 1**) by searching Pubmed and Web of Science from 2016 up to April 15^th^, 2020. We used the search terms “immune checkpoint inhibitors *” OR “immunotherapy *” AND “non-small cell lung cancer*” AND “pneumonitis*” with related terms including MeSH terms as well as keywords. All case reports were included. And we found that the mean time to CIP onset from the start of ICI therapy was approximately 10 weeks (2.5 months; **Table 2**). No difference was found in the median time from treatment to CIP onset between patients with improved/resolved CIP and deteriorated/maintained CIP (P=0.547) (**Table 2**). The onset of CIP reportedly occurred as early as hours to days—or as late as several months—after the first ICI dose; however, more severe CIP grades usually had onset within the first 100 to 200 days of ICI therapy (87). The median time to CIP onset was not related to disease severity (88), and onset seemed to occur earlier for patients treated with combination ICIs (18). Of note, CIP might develop months after therapy termination, which suggests that continuous vigilance after drug discontinuation is necessary (54).

**Table 1:** Published case reports and case series of immune checkpoint inhibitor-associated pneumonitis.

Author	Year	Patient	Country	Cancer Type	Histologic type	Genomic alterations (PD-1/PD-L1) (%)	Drug		Previous therapy	Time of onset	Grade of CIP	withdrew the drug	Time to withdrew the drug	Treatment	Outcome		
							PD-1 inhibitors	PD-L1 inhibitors							CIP	CIP course(weeks)	Other iAEs
Yin et al. (55)	2021	62/M	China	NSCLC	AC	55	pembrolizumab		chemotherapy	After 27 weeks	2	Yes	After 27 weeks	prednisolone	Improved	3	/
Shannon (9)	2020	62/F	USA	NSCLC	AC	/	pembrolizumab		radiotherapy	After 11 days	3	Yes	After 11 days	solumedrol	Improved	/	/
Shannon (9)	2020	82/M	USA	NSCLC	unknown	/	nivolumab		/	After 6 weeks	3	Yes	After 6 weeks	/	Deteriorated	/	/
Shannon (9)	2020	66/F	USA	NSCLC	unknown	/	pembrolizumab		/	After 6 weeks	2	Yes	After 6 weeks	steroid	Resolved	/	/
Shannon (9)	2020	69/M	USA	NSCLC	unknown	/	nivolumab		/	After 6 weeks	2	Yes	After 6 weeks	steroid	Improved	/	/
Davies et al. (56)	2020	68/M	USA	NSCLC	AC	1	pembrolizumab		chemotherapy	After 12 weeks	2	Yes	After 12 weeks	prednisone; PPI; TMP/SMX	Recurrent(8w)-Resolved	16	/
Utsumi et al. (57)	2020	59/M	Japan	NSCLC	unknown	1	pembrolizumab		radiochemotherapy	After 3 weeks	4	Yes	After 3 weeks	methylprednisolone; prednisolone; tacrolimus; cyclophosphamide	Deteriorated(1w)-Improved	8.4	/
Julie et al. (58)	2020	61/M	USA	NSCLC	unknown	1	pembrolizumab		radiochemotherapy	After 12 weeks	4	Yes	After 12 weeks	antibiotics; high dose steroids	Deteriorated	/	NSTEMI and CHF exacerbation
Wang et al. (59)	2020	55/M	China	NSCLC	AC	60	pembrolizumab		chemotherapy	After 24 weeks	3	Yes	After 24 weeks	methylprednisolone	Not completely resolved	3	/
Hirohide et al. (60)	2020	/	Japan	NSCLC	AC	55	pembrolizumab		radiochemotherapy	After 7 weeks	3	Yes	After 7 weeks	methylprednisolone	Improved	33	grade 1 typical radiation pneumonitis
Li et al. (61)	2019	57/M	China	NSCLC	unknown	60		atezolizumab	concurrent radio-chemotherapy; bevacizumab	After 5 days	3	Yes	After 5 days	antibiotics; methylprednisolone	Improved	/	thrombocytopenia, and cardiac dysfunction
Michael et al. (62)	2019	79/M	Austria	NSCLC	both	20	nivolumab		radiochemotherapy	After 8 weeks	3	Yes	After 8 weeks	antibiotics; corticosteroids; TMP/SMX	Deteriorated	/	/
Michael et al. (62)	2019	53/M	Austria	NSCLC	AC	70	nivolumab		surgery; radiochemotherapy	After 6 weeks	4	Yes	After 6 weeks	antibiotic; corticosteroid mycophenolate mofetil; TMP/SMX; ganciclovir	Deteriorated (8W)	/	/
Petri et al. (63)	2019	76/F	USA	NSCLC	AC	/	pembrolizumab		chemotherapy	After 12 weeks	4	Yes	After 12 weeks	antibiotics; methylprednisolone; prednisone; immunoglobulin	Improved	16	/
Eeden et al. (64)	2019	56/F	USA	NSCLC	unknown	/	nivolumab		radiochemotherapy	About 6 months	3	Yes	About 6 months	antibiotics; corticosteroids; antituberculosis treatment	Deteriorated	/	grade 2 diarrhea
Tonk et al. (65)	2019	72/M	The Netherlands	NSCLC	unknown	/		durvalumab	radiochemotherapy	During infusion of the first cycle	3	Yes	During infusion of the first cycle	clemastin; dexamethasone and acetaminophen; prednisolone; mycophenolic acid	Recurrent after 12w,51w, finally maintained	73.4	/
Blanchard and Bouchard (66)	2019	69/F	Canada	NSCLC	SC	40	pembrolizumab		chemotherapy	After 21 weeks	4	Yes	After 21 weeks	methylprednisolone; bronchodilators; azithromycin	Not completely improved	40.4	/
Dickey et al. (67)	2019	60/F	Austria	NSCLC	SC	75	pembrolizumab		radiotherapy	After 15 weeks	2	Yes	After 15 weeks	antibiotics; methylprednisolone; prednisone	Recurrent(3W)-not completely improved	3.4	thrombotic thrombocytopenic purpura
Sato et al. (68)	2019	62/M	Japan	NSCLC	AC	80	pembrolizumab		/	After 9 weeks	2	Yes	After 9 weeks	dexamethasone	Improved	/	bowel perforation with acute diffuse peritonitis
Maria et al. (69)	2019	72/M	Greece	NSCLC	SC	/	nivolumab		radiochemotherapy	After 15 weeks	2	/	After 15 weeks	prednisolone	Resolved	22	grade 2 colitis; hypercalcemia
Fan et al. (70)	2019	80/M	China	NSCLC	SC	50	nivolumab		chemotherapy	After 10 weeks	2	Yes	After 12 weeks	prednisolone	Resolved	12	Febrile neutropenia
Neal et al. (25),	2018	66/M	USA	NSCLC	unknown	70	nivolumab		radiochemotherapy	After 18 weeks	3	Yes	After 18 weeks	methylprednisolone; prednisone; infliximab	Recurrent (2w, 4w) for 2 times finally resolved	6.8	/
Neal et al. (25)	2018	63/F	USA	NSCLC	unknown	60	pembrolizumab		radiotherapy	After 48 days	4	Yes	After 48 days	antibiotics; methylprednisolone; infliximab, cyclophosphamide	Deteriorated (2w)	2	/
Corine et al. (71)	2018	58/F	USA	NSCLC	unknown	1	nivolumab		radiochemotherapy; bevacizumab	After 14 weeks	3	Yes	After 14 weeks	antibiotics; prednisone	Recurrent for several times (6.4; 12.9; 17.1) and finally resolved	38.6	/
Koyoma et al. (72)	2018	46/M	Japan	NSCLC	unknown	/	nivolumab		chemotherapy; bevacizumab	After 2 weeks	3	/	After 2 weeks	methylprednisolone; prednisolone	Improved	1	/
Koyoma et al. (72)	2018	59/M	Japan	NSCLC	unknown	/	nivolumab		chemotherapy; erlotinib; bevacizumab	After 2 weeks	3	/	After 2 weeks	prednisolone	Deteriorated-Improved	/	/
Foukas et al. (73)	2018	58/M	USA	NSCLC	SC	/	nivolumab		radiochemotherapy	After 4 weeks	3	Yes	After 4 weeks	antibiotics; prednisolone; TMP/SMX	Recurrent after 4w and improved	12	/
Li et al. (74)	2018	52/M	China	NSCLC	unknown	50	pembrolizumab		radiochemotherapy	After 9 weeks	2	Yes	After 9 weeks	prednisolone	Not completely improved	16	/
Eduard et al. (75)	2018	77/M	Spain	NSCLC	AC	85	nivolumab		chemotherapy	After 36 weeks	2	Yes	After 28 weeks	antibiotics; methylprednisolone; TMP/SMX	Resolved	3	nephritis, hepatitis
Akella et al. (76)	2018	80/F	USA	NSCLC	unknown	/	nivolumab		chemotherapy	After 10 months	2	Yes	After 10 months	methylprednisolone	Resolved	16.4	/
Jodai et al. (77)	2018	62/M	Japan	NSCLC	AC	/	nivolumab		chemotherapy	After 6 weeks	2	Yes	After 6 weeks	antibiotics; prednisolone	Improved	/	/
Li et al. (78)	2017	67/M	USA	NSCLC	SC	50	nivolumab		radiochemotherapy	After 4 weeks	3	Yes	After 6 weeks	antibiotics; corticosteroid	Deteriorated(5w)-Improved	13	/
Kanai et al. (79)	2017	71/M	Japan	NSCLC	AC	/	nivolumab		chemotherapy	After 16 weeks	3	Yes	After 16 weeks	prednisolone; cyclosporine A; methylprednisolone; infliximab	Deteriorated (8W)	10.4	/
Takeru et al. (80)	2017	82/M	Japan	NSCLC	unknown	/	nivolumab		radiochemotherapy	After 3 weeks	2	/	/	methylprednisolone	Improved	14.4	radiation pneumonitis 2months after radiation; steroid
Kato et al. (81)	2017	39/M	Japan	NSCLC	unknown	/	nivolumab		radiochemotherapy	After 4 days	2	Yes	After 4 days	prednisone	Recurrent(12w)-improved	28	/
Kenji et al. (82)	2017	74/F	Japan	NSCLC	unknown	/	nivolumab		chemotherapy; bevacizumab	After 3 days	3	Yes	After 3 days	methylprednisolone; prednisolone	Deteriorated	1.5	/
Kenji et al. (82)	2017	67/F	Japan	NSCLC	unknown	/	nivolumab		radiochemotherapy; erlotinib; bevacizumab	After 1 week	3	Yes	After 1 week	betamethasone; methylprednisolone	Improved	3.9	/
Kenji et al. (82)	2017	75/F	Japan	NSCLC	unknown	/	nivolumab		radiochemotherapy	After 5 days	3	Yes	After 5 days	methylprednisolone; cyclophosphamide	Not completely improved	2.7	/
Balaji et al. (83)	2017	73/M	USA	NSCLC	unknown	/	nivolumab		chemotherapy	After 4 weeks	2~4	Yes	After 10 weeks	prednisone; bronchodilators; TMP/SMX	Recurrent (3weeks; 5weeks; 9 weeks) - maintained	11.3	/
Balaji et al. (83)	2017	70/F	USA	NSCLC	unknown	/	nivolumab		surgery; chemotherapy; ipilimumab (3 mg/kg)	After 3 days	3	Yes	After 3 days	prednisone	Recurrent(9W)-Improved	13.3	/
Naqash et al. (84)	2017	53/F	USA	NSCLC	AC	0		atezolizumab	concurrent radiochemotherapy	After 7 weeks	2	Yes	After 7 weeks	prednisone; tocilizumab	Resolved	5.6	arthritis
Shibaki et al. (85)	2017	68/M	Japan	NSCLC	SC	/	nivolumab		radiotherapy	After 8 weeks	2	Yes	After 8 weeks	prednisolone	Improved	4	
Shibaki et al. (85)	2017	55/M	Japan	NSCLC	unknown	/	nivolumab		radiotherapy	After 24 weeks	2	Yes	After 24 weeks	prednisolone	Improved	4	
Gounant et al. (86)	2016	70/M	USA	NSCLC	SC	80	nivolumab		chemotherapy; necitumumab (anti-EFGR monoclonal antibody)	After 12 weeks	2	Yes	After 12 weeks	prednisone	Recurrent 20w later-finally resolved	23.4	grade 2 hyperthyroidism

NSCLC, non-small cell lung cancer; TMP/SM, trimethoprim/sulfamethoxazole; PPI, proton pump inhibitors; NSTEMI, non–ST-segment elevation myocardial infarction; CHF, Congestive heart failure.

**Table 2:** Baseline characteristics of the NSCLC cases with CIP according to the CIP outcome.

CIP outcome Mean ± SD/N (%)	Total	Improved/Resolved	Deteriorated/Maintained	P-value
**N**	44	34	10	
**Age**	65.23 ± 9.84	64.27 ± 9.83	68.40 ± 9.69	0.232
**Sex**				0.798
Female	14 (32.56%)	11 (33.33%)	3 (30.00%)	
Male	29 (67.44%)	22 (66.67%)	7 (70.00%)	
**Genomic alterations (%)**	45.90 ± 29.62	47.82 ± 29.60	37.75 ± 32.66	0.554
**Country**				0.195
USA	18 (40.91%)	13 (38.24%)	5 (50.00%)	
Japan	14 (31.82%)	12 (35.29%)	2 (20.00%)	
China	5 (11.36%)	5 (14.71%)	0 (0.00%)	
Austria	3 (6.82%)	1 (2.94%)	2 (20.00%)	
Canada	1 (2.27%)	1 (2.94%)	0 (0.00%)	
Greece	1 (2.27%)	1 (2.94%)	0 (0.00%)	
The Netherlands	1 (2.27%)	0 (0.00%)	1 (10.00%)	
Spain	1 (2.27%)	1 (2.94%)	0 (0.00%)	
**Grade of CIP**				**0.002**
Grade 2	18 (40.91%	18 (52.94%)	0 (0.00%)	
Grade 3	19 (43.18%)	13 (38.24%)	6 (60.00%)	
Grade 4	7 (15.91%)	3 (8.82%)	4 (40.00%)	
**Histologic type**				0.079
AC	12 (27.27%)	10 (29.41%)	2 (20.00%)	
SC	8 (18.18%)	8 (23.53%)	0 (0.00%)	
Both	1 (2.27%)	0 (0.00%)	1 (10.00%)	
Unknown	23 (52.27%)	16 (47.06%)	7 (70.00%)	
**ICIs**				0.548
PD-1 inhibitors	41 (93.18%)	32 (94.12%)	9 (90.00%)	
PD-L1 inhibitors	3 (6.82%)	2 (5.88%)	1 (10.00%)	
**Recurrence times**				0.325
0	34 (77.27%)	26 (76.47%)	8 (80.00%)	
1	6 (13.64%)	6 (17.65%)	0 (0.00%)	
2	2 (4.55%)	1 (2.94%)	1 (10.00%)	
3	2 (4.55%)	1 (2.94%)	1 (10.00%)	
**Dose of onset**	4.18 ± 3.80	4.26 ± 3.93	3.90 ± 3.51	0.793
**Time of onset**	10.14 ± 9.48	10.62 ± 10.12	8.53 ± 7.09	0.547
**Steroid initial dose(mg/d)**	425.29 ± 451.82	474.43 ± 475.24	196.00 ± 263.30	0.301
**Steroid initial dose groups(mg/d)**				0.222
Low-dose <60	5 (29.41%)	3 (21.43%)	2 (66.67%)	
Intermediate-dose 60-500	6 (35.29%)	5 (35.71%)	1 (33.33%)	
High-dose 501-1000	6 (35.29%)	6 (42.86%)	0 (0.00%)	
**Steroid initial dose(mg/kg/d)**	1.24 ± 0.58	1.15 ± 0.57	1.80 ± 0.28	0.149
**Steroid initial dose groups(mg/kg/d)**				0.177
Low-dose <1	8 (53.33%	8 (61.54%)	0 (0.00%)	
Intermediate-dose1-2	6 (40.00%)	4 (30.77%)	2 (100.00%)	
High-dose >2	1 (6.67%)	1 (7.69%)	0 (0.00%)	
**Steroid taper time**	10.46 ± 9.94	10.20 ± 10.13	12.00 ± 10.58	0.649
**Steroid course**	14.43 ± 15.14	13.46 ± 11.20	19.72 ± 30.35	0.404
**Antibiotics**				0.077
No	28 (63.64%)	24 (70.59%)	4 (40.00%)	
Yes	16 (36.36%)	10 (29.41%)	6 (60.00%)	
**Immunosuppressive drugs**				0.081
No	35 (79.55%)	29 (85.29%)	6 (60.00%)	
Yes	9 (20.45%)	5 (14.71%)	4 (40.00%)	
**OS**				**0.001**
Alive	20 (57.14%)	19 (73.08%)	1 (11.11%)	
Dead	15 (42.86%)	7 (26.92%)	8 (88.89%)	
**Survival weeks**	55.35 ± 46.26	61.44 ± 49.71	34.49 ± 23.88	0.168
**CIP Course (weeks)**	12.64 ± 14.20	11.66 ± 10.81	16.45 ± 23.93	0.402
**Clinical response**				**0.027**
Complete response	2 (5.71%)	2 (7.69%)	0 (0.00%)	
Partial response	6 (17.14%)	6 (23.08%)	0 (0.00%)	
Tumor progressed	5 (14.29%)	5 (19.23%)	0 (0.00%)	
Stable	7 (20.00%)	6 (23.08%)	1 (11.11%)	
Unknown	15 (42.86%)	7 (26.92%)	8 (88.89%)	

NSCLC, non-small cell lung cancer; CIP, checkpoint inhibitor pneumonitis; SC, squamous cell carcinoma; AC, adenocarcinoma; ICIs, immune checkpoint inhibitors; OS, overall survival.

Bold values: two-sided P-values less than 0.05 were considered to identify statistical significance.

## Potential Mechanism of CIP

In animal models with deficiencies of PD-1 and CTLA-4, animals exhibited lung infiltration (89, 90), which could clarify questions about how CIP develops (91). The potential mechanisms driving ICI-related pneumonitis are outlined in the following sections.

### Increased T-cell Activity Against Cross-Antigens

Enhanced and/or targeted T-cell activity against cross-antigens shared between tumor and normal tissues may result in irAEs (14, 91). Furthermore, cytotoxic antigen-directed T-cell responses may drive CIP pathogenesis. Significant lymphocytosis enriched with CD8+ T cells has been examined in the pulmonary tissues and BAL from patients with clinical typical CIP (92, 93). In NSCLC, Suresh et al. (94) noted that CD4+ T cells predominated in the BAL of patients with CIP. Notably, decreased expression of PD-1 and CTLA-4 and increased numbers of central memory T cells were observed within the regulatory T-cell population, which suggested that dysregulation of T cells may result from activation of pro-inflammatory immune subsets (alveolar T cells) and weakening of the anti-inflammatory regulatory T-cell phenotype.

In addition, Laubli et al. (95) conducted T-cell receptor sequencing on tumor-infiltrating lymphocytes and T cells infiltrating the inflammatory CIP lesions and found a notable overlap of T-cell repertoire in these sites but not in the secondary lymphoid organs or peripheral blood. Despite the indeterminant nature of antigen specificity, these data highlighted the cytotoxic effects of T cells on the instigation of CIP. Moreover, the predictive value of tumor-infiltrating lymphocytes has been illustrated in meta-analyses (96, 97). An elevated level of CD4+/CD8+ T-cell infiltration in the malignant cells showed superior outcomes in survival. However, an increasing number of FOXP3+ regulatory T cells, a subtype of CD4+ T cells with immunosuppressive actions, was associated with poor survival. These results have been reported from patients with ICI-related pneumonitis, and more evidence is needed from future studies to explore CIP mechanisms.

### Increased Level of Autoantibodies and Inflammatory Cytokines

Pre-existing autoantibodies potentially linked to the development of irAEs in NSCLC, such as anti–thyroid peroxidase antibodies, anti-thyroglobulin antibodies, antinuclear antibodies, anti–rheumatoid factor antibodies, have been explored in recent studies (98). Tahir et al. (99) performed a mass screening of autoantibodies in patients who underwent ICI therapy by using high-throughput serological analysis of recombinant cDNA expression (i.e. SEREX). They identified an elevated plasma level of anti-CD74 from two patients with CIP in a discovery cohort and subsequently verified a 1.34-fold increase from 10 patients with CIP in a confirmation cohort. Intriguingly, samples of viral-mediated interstitial pneumonitis have also displayed an overexpression of CD74 (100), presenting a pathogenic nidus for CIP development. However, the specific antibodies associated with CIP should be prioritized for exploration. In terms of inflammatory cytokines, case reports of severe CIP have identified some cytokines linked to the appearance of CIP. Interleukin-6 (IL-6), IL-17A, IL-35, C-reactive protein (CRP), procalcitonin (PCT), surfactant protein-D (SP-D), and Krebs von den Lungen-6 (KL-6) were reportedly more common in patients with NSCLC who developed CIP than in those without CIP (25, 52, 57, 82, 84). In particular, SP-D and KL-6 reflected alveolar epithelial cell injury. All these cytokines also broadly serve as biomarkers for adverse events caused by ICIs.

### Enhanced Complement-Mediated Inflammation

The function of complement-mediated inflammation may be enhanced by the direct combination of anti–CTLA-4 with CTLA-4 located on benign tissues, including the pituitary gland (14, 91). This mechanism may explain why pituitary inflammation could be a specific irAE of anti–CTLA-4 antibodies (101). Although CIP is more frequently observed with PD-1/PD-L1 blockades than with CTLA-4 blockades (102), CIP has not yet become a symbolic irAE of anti–PD-1/PD-L1 antibodies. After a review of the relevant literature, we speculate that the major causes of CIP may be the first two mechanisms described before. Additional exploration is required to deepen our understanding of CIP in NSCLC.

## Risk Factors of CIP

Current evidence from retrospective studies and case reports has identified many potential risk factors for ICI-related pneumonitis (6, 24, 53, 72, 103–105). These include baseline patient characteristics, disease features, and therapy management. Specific factors include age, sex, smoking status, previous lung disease, tumor histological type, PD-1 blockade, combination therapy, and prior RT.

### Baseline Patient Characteristics

The influence of age on the response to immunotherapy has not been studied comprehensively or systematically. Cho et al. (28) found that patients who had CIP were often older than 70 years (54.5% of total population studied, P=0.025). However, other literature has suggested that older age would not adversely relate to rates of toxicities or therapeutic response to ICI therapies (106, 107). A retrospective study recruited 205 patients with NSCLC and reported a higher incidence of CIP in women than in men, though the difference was not significant (24). Similarly, in another study, former or current smokers developed CIP more often than nonsmokers (P=0.03) (108). The evidence must be verified, but it does offer a new direction for continued research (87).

### Disease Features

Pre-existing pulmonary diseases, including interstitial lung disease (ILD), chronic obstructive pulmonary disease (COPD), asthma, pneumothorax, pleural effusion, and pulmonary fibrosis, have been closely associated with the development of CIP in patients with NSCLC (19, 27, 29, 49, 50). The incidence of ICI-related pneumonitis in patients with pre-existing ILD was approximately three times higher than in those without ILD (29% vs 10%, P=0.027) (49). Patients with asthma and COPD were more likely to develop CIP (2.3% higher incidence vs those without COPD) (27). Notably, Nicholas et al. (29) found increasing numbers of lymphocytes dominated by CD4+/CD8+ T cells and high PD-L1 expression in the lungs of patients with NSCLC who had COPD, which might suggest longer PFS in patients receiving ICIs without COPD. A case-control study that included patients with pneumothorax, pleural effusion, and pulmonary fibrosis found a high risk of CIP in these patients but noted a low mortality rate and a high remission rate in the same group after treatment with corticosteroids (104). With regard to the tumor type, subgroup analyses of previous research showed that patients with the SCC subtype of NSCLC experienced a greater occurrence of CIP, but a lower mortality rate, compared with those diagnosed with the AC subtype (5, 10, 11, 21, 24, 37, 38, 41).

### Therapy Management

RT reportedly has a synergistic effect with immunotherapy (14, 23). Intriguingly, RT itself could induce radiation pneumonitis in more than 30% of patients (109). Even when the radiation pneumonitis resolves, patients may present with severe radiation recall pneumonitis after treatment with ICIs (60). The Keynote-001 trial (110) explored the clinical efficacy of PD-1 inhibitors in patients with NSCLC and found a higher incidence of any-grade CIP in patients who received RT before ICI therapy (pembrolizumab, 13%) compared with those who did not receive RT (1%, P<0.05). The timing of RT and ICI use must be studied and discussed in more detail, whether a shorter interval between the two treatments could increase mutual toxicity or not remains unclear. The PACIFIC trial (111) compared CIP rates according to the initial time to start durvalumab after chemoradiotherapy (within 14 days or between 14 and 56 days) and found that the earlier start time did not increase the risk of CIP. RT parameters that may influence the development of CIP have also been studied, dosimetric parameters of prior chest RT, courses, timing, and technique were not considered significant risk factors for CIP development (48).

Monotherapy and combination therapy with ICIs appear to have distinct incidences of CIP in NSCLC. With ICI monotherapy, use of PD-1/PD-L1 inhibitors instead of CTLA-4 inhibitors increased the risk of CIP development (64). A meta-analysis (87) that included 19 trials found that PD-1 blockade treatment was associated with a statistically significantly higher incidence of CIP than PD-L1 blockade (3.6% vs 1.3%, P=0.001). In addition, the analysis reported no significant difference in the incidence of CIP in patients who received pembrolizumab or nivolumab. However, Fukihara et al. (47) found that more patients treated with pembrolizumab than with nivolumab developed CIP (63% vs 37%, P=0.004). Moreover, the incidence of CIP in patients treated with combination therapy increased twofold to threefold compared with patients treated with monotherapy (30, 87). The need for antibiotics and immunosuppressive drugs (112) were also predominant risk factors for pulmonary infection after ICIs.

## Manifestations of CIP

### Clinical Manifestations

The main clinical symptoms of CIP are relatively nonspecific and usually are similar to certain forms of ILD (23). CIP is characterized by fever, cough, chest pain, shortness of breath, dyspnea, fatigue, or respiratory failure (104). Bloody sputum or hemoptysis, hypotension, tachycardia or palpitation, diarrhea, and joint pain are less common (**Supplemental Table 1**). In our analysis, dyspnea accounted for the most significant symptom of CIP (63.64%), followed by cough (36.36%) and fever (25.00%). Rashes were also commonly reported. Crackles on thorax auscultation manifested only in more advanced-grade CIP (23, 86).

### Imaging Manifestations

**Figure 2:**
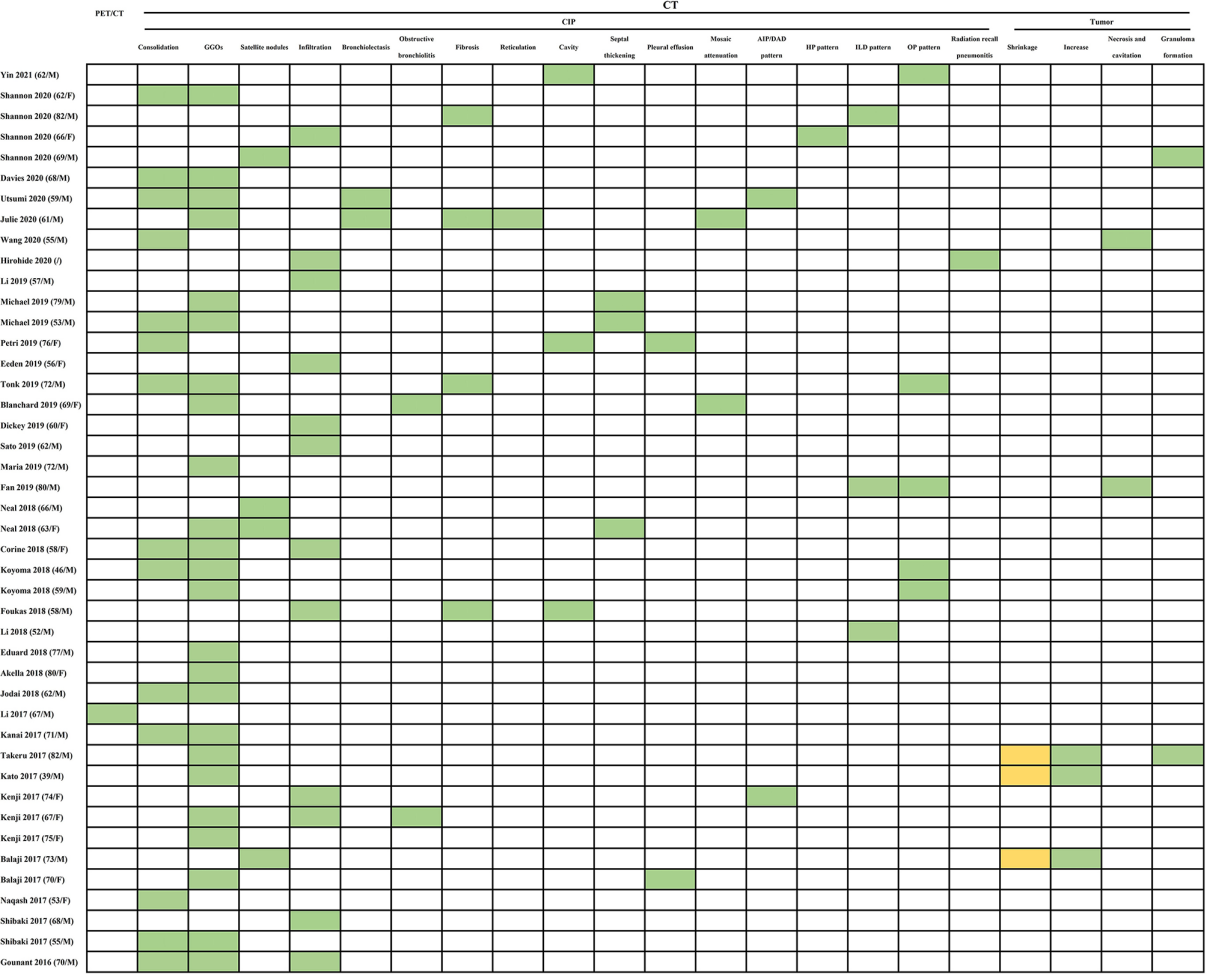
Summarized results of radiological tests for diagnosis in published cases reports/case series. The radiological tests include PET/CT and CT findings. PET/CT, positron emission tomography/computed tomography; CT, computed tomography; GGOs, ground-glass opacities; AIP/DAD, Acute interstitial pneumonia (AIP)/diffuse alveolar damage (DAD); HP, hypersensitivity pneumonitis; ILD, interstitial lung disease; OP, organizing pneumonia. Abnormal: green grid; normal: orange grid; undone: white grid.

As awareness and experience with CIP increase among researchers, large-scale studies have categorized the various radiologic patterns. Acute interstitial pneumonia (AIP)/acute respiratory distress syndrome (ARDS)/diffuse alveolar damage (DAD), cryptogenic organizing pneumonia (OP), ground-glass opacities (GGOs), nonspecific interstitial pneumonia, hypersensitivity pneumonitis (HP), bronchiolitis, radiation recall pneumonia, and an unclassified type have been recognized as subtypes of CIP according to imaging features in several studies concerning NSCLC (5, 6, 18, 20, 23, 88, 113). These different radiographic patterns of CIP could also be described as a spectrum of the pulmonary injury evolution process, from the acute stage (AIP/ARDS/DAD) to the organizing stage (OP) and fibrotic stage (nonspecific interstitial pneumonia) (5, 6). The GGOs and consolidation (**Figure 2**) non-segmentally distributed in the dominant lung or bilaterally opposite the tumor, which have been considered typical computed tomography (CT) features in CIP of NSCLC (104, 113), represented 54.55% (24/44) and 31.82% (14/44), respectively, of the CT presentations in our analysis.

The OP pattern was the most common pattern for CIP in NSCLC (6, 113). The common manifestation of the OP pattern was bilateral peribronchovascular and subpleural GGOs, predominately in the middle to lower lung (113). Reversed atoll or halo sign, a circumferential consolidation surrounding an interior area of mosaic (ground-glass) attenuation, has been considered a relatively specific characteristic for OP in CIP (114). In addition, peribronchovascular pulmonary nodules smaller than 10 mm have been depicted in the OP pattern (113). However, the nodules could be mass-like with spiculated margins (115) or could be a peritumoral shadow (80, 104), reflecting obscure presentations of the tumor. This phenomenon has been regarded as pseudoprogression of malignancy (80, 104, 115). Two cases (80, 81) that we included presented with GGOs associated with an increase in tumor size (pseudoprogression). Pseudoprogression could be distinguished from CIP by evaluation of serum markers (carcinoembryonic antigen, cytokeratin fragment) (116) and by bronchoscopic narrow-band imaging and biopsy (117).

The nonspecific interstitial pneumonia pattern was the second most frequently reported pattern of CIP (113). It commonly manifests with GGOs and reticulation in the lower lobe of the lung (118). The specific finding was described as a subpleural sparing of the dependent and posterior lower lobe of the lung (115). Conversely, the HP pattern was a relatively uncommon radiologic abnormality of CIP. Centrilobular or diffuse GGOs with the predominance of mid-to upper-lobe location were the radiologic features of the HP pattern (113). This pattern can be distinguished from an HP pattern related to allergen exposure by obtaining definite patient histories about occupational and other exposures. The AIP/ARDS/DAD pattern exhibited the most severe extent of pulmonary involvement on imaging, presenting with diffuse or patchy GGOs or consolidation with involvement in the majority or all of the lung. This presentation often exhibits a “crazy-paving” pattern and interlobular septal thickening (115). Bronchiolitis has been found only in one retrospective cohort study and a few case reports (66, 86, 88, 118). Typically, it appears as a tree-in-bud pattern in the region of centrilobular nodularity. However, even bronchiolitis may be investigated as a distinct CIP pattern without infectious symptoms.

Radiation recall pneumonia is an inflammatory reaction that occurs in previously irradiated regions after exposure to some inciting agents; it manifests as consolidation and GGOs limited to the previously radiated area. Possible mechanisms of this type of pneumonia include stem cell function changes in the irradiated field triggered by hypersensitivity reactions to an idiosyncratic drug (119). Some case reports have presented radiation recall pneumonia in patients with NSCLC after treatment with ICIs (60, 85). Patients who receive RT and develop new pulmonary changes demarcated from the adjacent lung in the initial radiation field should be preferentially suspected of having radiation recall pneumonia.

### Pathological Manifestations

**Figure 3:**
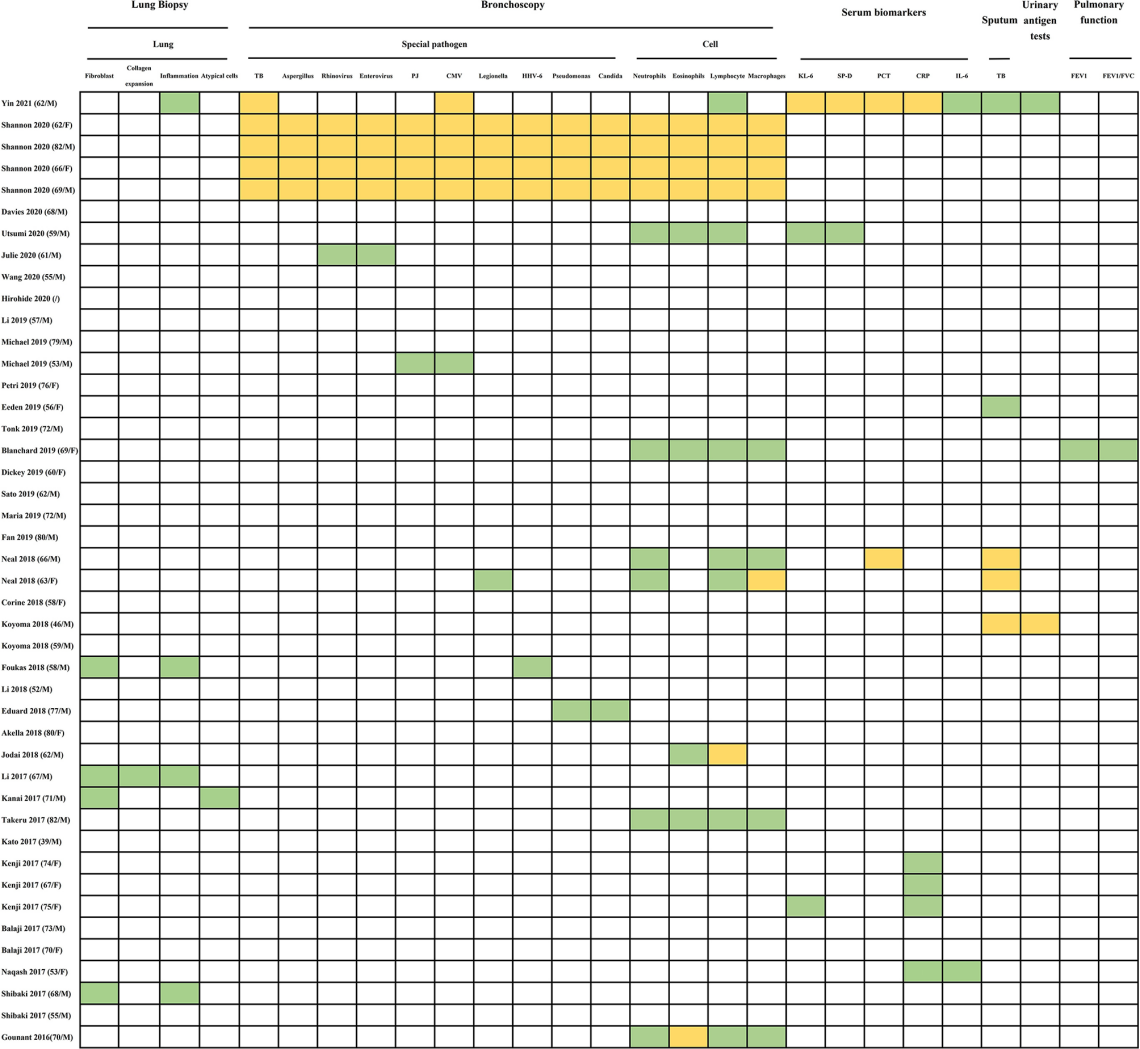
Summarized results of histological, laboratory and pulmonary function tests for diagnosis in published cases reports/case series. The histological test includes lung biopsy. The laboratory tests include BAL (special pathogen and cells) in bronchoscopy, serum, sputum, urinary antigen test. The pulmonary function tests include FEV1 and FEV1/FVC. TB, tuberculosis; PJ, pneumocystis jirovecii; CMV, cytomegalo-virus; HHV-6, human herpes virus 6; KL-6, krebs von den Lungen-6; SP-D, surfactant protein-D; PCT, procalcitonin; CRP, C-reactive protein; IL-6, interleukin-6; FEV1, forced expiratory volume in one second; FEV1/FVC, fractional volume change. Abnormal: green grid; normal: orange grid; undone: white grid.

Not all patients with CIP will receive lung biopsy, especially in patients with ICI-related ILD. In our analysis, only 5 of 44 patients were considered for this examination (**Figure 3**). Lung biopsies may increase the risk of acute deterioration in ILD and may not obtain definite histologic types if the harvested specimen is small. However, transbronchial lung biopsy could rule out alternative etiologies during the differential diagnosis. Literature reports have provided a limited pathological pattern of CIP, with a range of different presentations that includes OP, DAD, eosinophilic pneumonia, cellular interstitial pneumonitis, and nonspecific or granulomatous inflammation (6, 18, 88). The interstitial inflammatory infiltration might include elevated levels of eosinophil, poorly formed granulomas, and lymphocytes (18). The cases that we included specifically mentioned the pathological manifestation of alveolar parenchyma with fibroblast foci (four cases) (73, 78, 79, 85), mild collagen expansion of the alveolar septa (one case) (78), nonspecific chronic inflammation (four cases) (73, 78, 85), and atypical cells (one case) (79).

## Diagnosis of CIP

Because specific clinical or radiologic markers are absent, diagnosis of CIP is quite difficult. CIP is typically a diagnosis of exclusion, one that should rule out infection, tumor progression, and radiation-related pneumonitis (25). New emerging or the deterioration of respiratory symptoms—especially dry cough, dyspnea, and decreasing oxygen saturation—after ICI therapy for NSCLC require consideration of CIP (23). The diagnostic workup (**Figure 3**) to identify an etiology should include tests for a source of infection (including nasal swab, sputum/urine culture, and blood culture); tests for special pathogens (fungus, tuberculosis spot test); chest radiography (high-resolution CT); and bronchoscopy with BAL (17, 18, 20). Lung biopsy is not mandatory, and both drugs and infectious history can occasionally help to interpret results. Utilization of diagnostic tests is related to the suspected pneumonitis grade (6).

Common differential diagnoses for CIP include pulmonary infections, pulmonary embolism, DAD, lung cancer with underlying progression, cancerous lymphangitis, pulmonary interstitial edema caused by heart failure, fulminant myocarditis (120), and RIP (5, 23). Opportunistic pulmonary infections, including tuberculosis (TB) pneumonia, *aspergillosis*, *cytomegalovirus* pneumonia (CMVP), and *Pneumocystis jirovecii* pneumonia (PJP), have been the foremost differential diagnoses for CIP in the NSCLC population (26, 121–125). Inthasot et al. (26) reported two cases of severe lung infections complicating the treatment of nivolumab for NSCLC and emphasized the importance of eliminating the possibility of opportunistic infections. Notably, ICIs could cause special pathogen infections in some patients through induction of CIP. We included several cases of patients who developed CIP during ICI therapy and consequently developed rhinovirus/enterovirus (58), CMVP (62), PJP (62), *legionella* (25), human herpesvirus 6 (HHV-6) (73), *pseudomonas*, or *candida* (75) infections (**Figure 3**). Because ICIs activate tumor immunity by inhibiting PD-1/PD-L1/CTLA-4, they might also simultaneously inhibit immunity to infection. Although the infections we described here as differential diagnoses are not usually categorized as drug-induced pneumonias, we included this series of reports to exemplify challenges in differentiating intensified infection from drug-induced pneumonia. Since from a drug safety perspective, the infection did lead to a few deaths.

The CT manifestations of CIP in patients with pulmonary AC sometimes resembled those of interstitial pneumonitis (126), especially of the OP pattern. Ichikawa et al. (127) reported that 2% of patients (13/564) with resected pulmonary AC presented with an OP pattern. Kanai et al. (79) reported a case of coexisting CIP and tumor invasion, which complicated the diagnosis and management of the lung disease. Aggressive lung biopsy was recommended in that study to correctly diagnose CIP in patients with NSCLC that mimicked the OP pattern or existed the tumor invasion.

RIP, an early lung injury induced by radiation, is also a difficult differential diagnosis in CIP. The approximate onset (1 to 3 months), similar imaging features (GGOs and diffuse haziness), and shared pathological feature (lymphocytic alveolitis) increased the level of challenge in distinguishing CIP from RIP (128–130). However, a distinct lesion location may assist in finding the difference between the two. RIP mainly exists in the radioactive region, and CIP mostly occurs outside the RT fall-off dose or in the low-dose field (48). Interestingly, both CIP and RIP have the same first-line therapy (corticosteroids) (121, 128). Meanwhile, radionics has emerged as a new approach to predict CIP by automatically extracting radiologic features for synthesis analysis (131).

In summary, CIP requires a precise diagnosis, including grade assessment, and monitoring of CIP requires a multidisciplinary method. Such monitoring often involves infectious disease specialists, pathologists, radiologists, pulmonologists, and cardiologists (121).

## Management of CIP

CIP is deemed a self-limiting disease. No prospective trials, to our knowledge, have evaluated the optimal therapeutic modality for CIP (5, 24). Current guidelines for CIP, therefore, recommended corticosteroids as the primary therapy approach (121, 132, 133). These decisions are based on the strength of case reports and clinical experience (5, 24). Different definitions of CIP grades are shown in **Supplemental Table 2** (121, 133). Clinical improvement is usually observed after 48 to 72 hours of corticosteroid use, and patients without regression of CIP-related symptoms have been considered steroid refractory and treated with immunosuppressive agents (121, 133).

For patients with grade 1 CIP, clinical symptoms, imaging changes, and pulmonary function (diffusing capacity and spirometry) should be closely monitored for 3 to 4 weeks (122, 123, 134, 135). Tentatively stopping ICI treatment can be considered reasonable for mild cases of CIP (23). When the condition worsens, though, interruption of the ICI should be combined with initiation of low-dose steroids (0.5 to 1 mg/kg/d) (9, 136).

**Figure 4:**
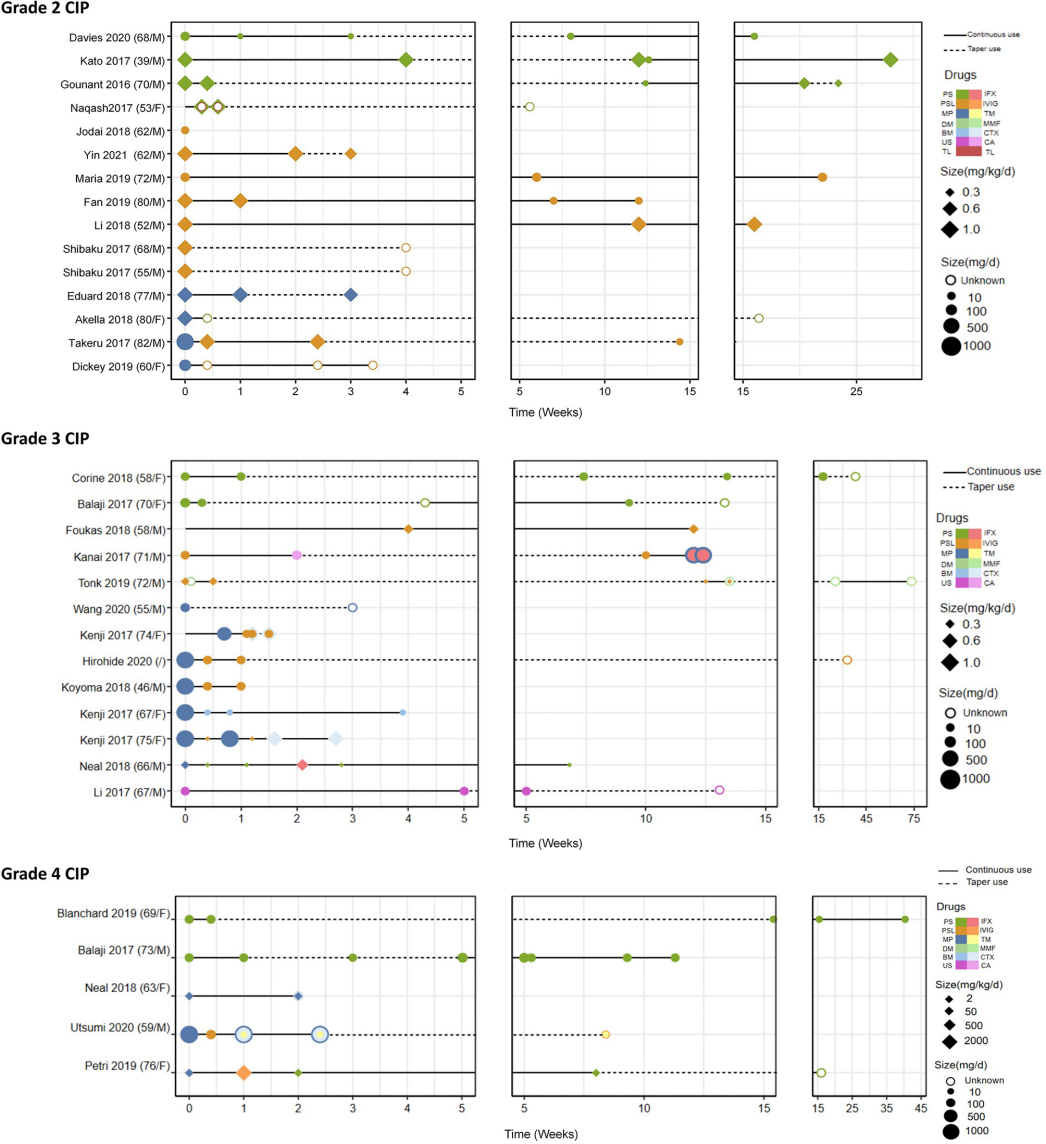
The steroid therapy including every drug that every case utilized and the definite continuous and taper time. PS, prednisone; PSL, prednisolone; MP, methylprednisolone; DM, dexamethasone; BM, betamethasone; US, unspecific; TL, tocilizumab; IFX, infliximab; IVIG, immunoglobulin; TM, tacrolimus; MMF, mycophenolate mofetil or mycophenolic acid; CTX, cyclophosphamide; CA, cyclosporine A.

For patients with grade 2 CIP, withholding the ICIs and beginning intermediate-dose steroids (1 to 2 mg/kg/d) followed by a taper by 5 to 10 mg/week for 4 to 6 weeks have been proposed (133). In our analysis, we summarized the management characteristics stratifed by CIP grade (**Table 3**) and listed every drug that every case used (**Figure 4**). We converted the different steroid doses to methylprednisolone (MP) equivalents and divided these into three groups (low-dose, intermediate-dose, and high-dose groups) according to the initial equivalent administered at the beginning of the therapy. We also noticed that some cases did not describe the weight of patients, which led to two different specifications of steroid dose (mg/d and mg/kg/d). In patients with grade 2 CIP (**Table 3**), 60% of patients were administered intermediate-dose steroids (60 to 500 mg/d). In other cases, 80% of patients with grade 2 CIP started with low-dose steroids (< 1 mg/kg/d). In addition, bronchoscopy and/or BAL plus initiation of empirical antibiotics when infection is suspected are recommended (14, 137). If clinical improvement does not happen after 2 to 7 days of monitoring, increasing the corticosteroid dose and adding immunosuppressive drugs should be considered (121, 138). Restarting ICI therapy may be considered when CIP is stable, has improved to grade ≤ 1, or has improved with 10 mg/d of prednisone (23). After re-initiation, physicians should evaluate clinical indicators every 3 days and perform chest imaging once a week to monitor for the flare and recurrence of CIP (9).

**Table 3:** The characteristics related to management of CIP stratified by grade of CIP.

Grade of CIP Mean ± SD/N (%)	Total	Grade 2	Grade 3	Grade 4	P-value
**N**	44	18	19	7	
**Steroid initial dose (mg/d)**	425.29 ± 451.82	280.40 ± 411.75	527.56 ± 469.29	360.00 ± 554.26	0.426
**Steroid initial dose groups (mg/d)**					0.379
Low-dose <60	5 (29.41%)	1 (20.00%)	2 (22.22%)	2 (66.67%)	
Intermediate-dose 60-500	6 (35.29%)	3 (60.00%)	3 (33.33%)	0 (0.00%)	
High-dose 501-1000	6 (35.29%)	1 (20.00%)	4 (44.44%)	1 (33.33%)	
**Steroid initial dose (mg/kg/d)**	1.24 ± 0.58	0.86 ± 0.10	2.00 ± 0.40	2.00 ± 0.00	**<0.001**
**Steroid initial dose groups (mg/kg/d)**					**0.007**
Low-dose <1	8 (53.33%)	8 (80.00%)	0 (0.00%)	0 (0.00%)	
Intermediate-dose 1-2	6 (40.00%)	2 (20.00%)	2 (66.67%)	2 (100.00%)	
High-dose >2	1 (6.67%)	0 (0.00%)	1 (33.33%)	0 (0.00%)	
**Steroid taper time**	10.46 ± 9.94	7.20 ± 5.35	16.37 ± 14.60	8.25 ± 4.79	0.154
**Steroid course**	14.43 ± 15.14	12.23 ± 8.54	16.35 ± 20.75	15.62 ± 14.75	0.776
**Immunosuppressive drugs**					**0.016**
No	35 (79.55%)	17 (94.44%)	15 (78.95%)	3 (42.86%)	
Yes	9 (20.45%)	1 (5.56%)	4 (21.05%)	4 (57.14%)	
**Antibiotics**					**0.011**
No	28 (63.64%)	14 (77.78%)	13 (68.42%)	1 (14.29%)	
Yes	16 (36.36%)	4 (22.22%)	6 (31.58%)	6 (85.71%)	
**Recurrent times**					0.312
0	34 (77.27%	14 (77.78%)	14 (73.68%)	6 (85.71%)	
1	6 (13.64%)	4 (22.22%)	2 (10.53%)	0 (0.00%)	
2	2 (4.55%)	0 (0.00%)	2 (10.53%)	0 (0.00%)	
3	2 (4.55%)	0 (0.00%)	1 (5.26%)	1 (14.29%)	
**CIP outcome**					**0.003**
Improved/Resolved	34 (77.27%)	18 (100.00%)	13 (68.42%)	3 (42.86%)	
Deteriorated/Maintained	10 (22.73%)	0 (0.00%)	6 (31.58%)	4 (57.14%)	
**CIP course (weeks)**	12.64 ± 14.20	10.30 ± 7.90	14.85 ± 19.20	12.96 ± 13.09	0.673
**OS**					**0.019**
Alive	20 (57.14%)	12 (85.71%)	5 (35.71%)	3 (42.86%)	
Dead	15 (42.86%)	2 (14.29%)	9 (64.29%)	4 (57.14%)	
**Survival time (weeks)**	55.35 ± 46.26	72.92 ± 58.13	41.00 ± 29.64	46.00 ± 37.33	0.198
**Clinical response**					**0.018**
Complete response	2 (5.71%)	0 (0.00%)	2 (14.29%)	0 (0.00%)	
Partial response	6 (17.14%)	6 (42.86%)	0 (0.00%)	0 (0.00%)	
Tumor progressed	5 (14.29%)	2 (14.29%)	2 (14.29%)	1 (14.29%)	
Stable	7 (20.00%)	4 (28.57%)	1 (7.14%)	2 (28.57%)	
Unknown	15 (42.86%)	2 (14.29%)	9 (64.29%)	4 (57.14%)	

CIP, checkpoint inhibitor pneumonitis; OS, overall survival.

Bold values: two-sided P-values less than 0.05 were considered to identify statistical significance.

For patients with grade 3 to 4 CIP, ICI therapy should be discontinued immediately and permanently. The initial doses of steroids (1 to 2 mg/kg/d and 2 to 4 mg/kg/d) were approved and included in guidelines by the American Society of Clinical Oncology guidelines and the European Society for Medical Oncology (15, 121), respectively. However, no clinical trials have identified optimal corticosteroid doses or durations; therefore, therapy duration has always been adjusted largely on the basis of response to steroid treatment. Our analysis showed that patients with grade 3 or 4 CIP most often received glucocorticoid pulse therapy (44% of patients with grade 3 and 33% of patients with grade 4; **Table 3**). Initial steroid dosages of 1 to 2 mg/kg/d were mostly used in patients with severe CIP (**Table 3**), and this dosage was consistent with the recommendations of the American Society of Clinical Oncology. Institutionally, we continue at the initial dosage until patients improve or remain stable (usually 1 week), at which time corticosteroids can be very slowly tapered during at least 5 to 8 weeks (9). Our data showed that the mean duration of a steroid taper was nearly 10 weeks, and the longest duration was in patients with grade 3 CIP (mean ± standard deviation of 16.37 ± 14.60 weeks) (**Table 3**). Additional immunosuppressants, including infliximab (IFX), mycophenolate mofetil, intravenous immunoglobulin, tacrolimus, ciclosporin (57, 79), and cyclophosphamide, should be considered when the symptoms do not regress after 48 to 72 hours of treatment with corticosteroids (6, 14, 23, 137). Empirical antibiotics may be used to prevent opportunistic infection (122, 139–141). Our data also showed that the rates of immunosuppressive drug use (grade 2: 5.56%, grade 3: 21.05%, grade 4: 57.14%, P=0.016) and antibiotic use (grade 2: 22.22%, grade 3: 31.58%, grade 4: 85.71%, P=0.011) gradually increased with increasing severity of CIP (**Table 3**).

Moreover, it has been reported that nearly one-fourth to one-third of patients experience CIP flares or recurrence after rapid corticosteroid tapers and appear recalcitrant to corticosteroid treatment (5). CIP recurrence may occur early in patients with more severe grade (grade 3 or 4) initially and have occurred most often in patients whose therapeutic course was shorter than 5 weeks (71, 142). The lengths of steroid courses from our data varied from 1 week to 73.4 weeks, and the mean duration for grade ≥ 2 CIP was more than 10 weeks (**Tables 2** and **3**). However, in patients whose steroid course was shorter than 5 weeks (25, 49, 55, 59, 67, 72, 75, 82), two patients (25, 67) experienced CIP recurrence. The highest CIP recurrence rate, 22.22%, occurred in patients with grade 2 CIP (**Table 3**). In addition, the steroid courses were centrally distributed in the first 5 weeks (**Figure 4**), which suggests that the changes to steroid dosages (in grade 2 CIP) and drugs (in grade 3 or 4 CIP) usually occurred in this window.

Current experience with immunosuppressive drugs to treat CIP is based mostly on extrapolation from data about their use to treat other irAEs, which lacked pathophysiological evidence (5). IFX and cyclophosphamide have been approved to treat ICI-related digestive toxicities, especially colitis (133, 143, 144). However, IFX could itself cause ILD and liver injury (145–147). In addition, it could weaken the ongoing anticancer immune activity initially launched by ICI treatment (25); this hypothesis is consistent with a prior study (18), which reported that half of patients with grade 3 CIP died despite receiving additional immunosuppressive drugs. As a second-line drug, mycophenolate mofetil remains controversial because of its suppressive effects on the T-cell response (148). IL-17 blockade reportedly relieved ICI-related gastrointestinal and skin irAEs (149). Current guidelines also recommend cyclophosphamide, mycophenolate mofetil intravenously (1 g twice daily), or IFX (5 mg/kg) as supportive care (121, 133, 135) for steroid-resistant patients with irAEs. Intravenous immunoglobulin was effective in ICI-mediated myasthenia gravis and did not blunt infection responses (150). Thus, intravenous immunoglobulin could become a logical choice for treating CIP in patients with suspected comorbid infections (24). Tocilizumab, an IL-6 inhibitor, has been used to treat rheumatologic irAEs (84). A case report showed that a patient with NSCLC and CIP experienced significant symptom relief after additional therapy with tocilizumab (151). However, whether tocilizumab should be included as an option in the second-line drugs to treat steroid-refractory patients with irAEs remains undetermined, because that approach lacks a comparison with other second-line drugs.

## Prognosis of CIP

**Figure 5:**
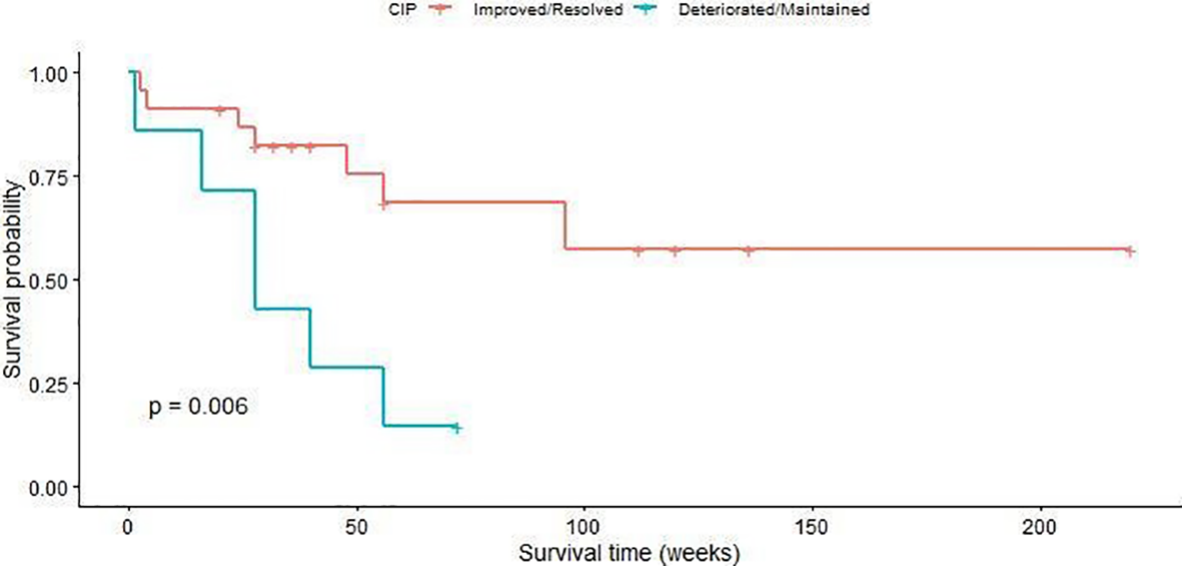
Overall survival curves of patients with checkpoint inhibitor pneumonitis.

Most studies have found that patients with CIP, especially with lower-grade disease, could see symptoms improve or resolve if they received corticosteroid therapies (18, 152). Similarly, our data (**Table 3**) demonstrated that patients with grade 2 CIP all experienced improvements in or resolution of CIP and had the highest OS (85.71%) versus patients with grade 3 or 4 CIP (OS of 35.71% or 42.86%, respectively, P=0.019). In addition, nearly half of patients with grade 2 CIP experienced a partial tumor response, whereas most patients with grade 3 or 4 CIP experienced tumor progression or maintenance. However, a single-center study (20) recently reported poor prognoses in patients with NSCLC who developed CIP. Suresh et al. (45) demonstrated that the ICIs did not significantly influence the short-term survival (disease control rate, overall response rate, or PFS) but did affect OS which decreased by 10 months in patients with CIP. Fukihara et al. (47) came to a similar conclusion regarding the decrease in OS. Patients with CIP (8.7 months) had a shorter OS after PD-1 blockade compared with those without CIP (23.0 months, P=0.015). We also evaluated the association between CIP and OS (**Figure 5**), and we found that patients who experienced deteriorated or maintained CIP were significantly more likely to have a poor prognosis compared with patients who experienced improved or resolved CIP (P=0.006). One potential reason might be that patients with CIP were more likely to be forced to quit ICI therapy to avoid lethal respiratory failure. Moreover, as a result of deteriorating physical status, abrasive pulmonary symptoms, and prolonged steroid management for CIP, patients with CIP tended to reject—and their physicians were more likely to hesitate or delay commencement or continuation of—aggressive anti-tumor treatment.

Recurrent phenomena related to the management of CIP have been explored in patients with NSCLC who received ICI therapy. These phenomena included recurrent pneumonia after completion of a steroid taper with or without restarting immunotherapy. Reports of reusing ICI therapy mainly occurred in patients with grade 1 or 2 CIP initially, since patients identified with grade 3 or 4 CIP generally withdrew treatment permanently (47). The reported recurrence ratio after reusing immunotherapy varied from 17% to 30% (18, 55, 122). Our analysis (**Table 2**) showed that the overall recurrence ratios with and without re-challenge ICI therapy were 6.82% (3/44) and 22.73% (10/44), respectively. Among three patients (65, 73, 81) who re-challenged ICI therapy after clinical regression of CIP, two experienced recurrence after restarting ICIs (65, 81), and one patient successfully improved by discontinuing immunotherapy and beginning treatment with antibiotics and steroids (73). Recurrent pneumonitis severity, location of involvement, and pattern might vary compared with the initial manifestation of CIP.

Predictive factors for CIP are still under investigation. Currently, the exploration of serum markers, cytokines/chemokines, and cellular biomarkers have interested clinicians (137). Increased carcinoembryonic antigen (CEA) serum levels reportedly relate to both tumor progression and the simultaneous regression of recurrent CIP (153), which represents an early association with both durable toxicity and durable response. In addition, a low level of serum albumin was an independent predictor of CIP in patients with NSCLC (odds ratio=0.381, 95% CI=0.179–0.808, P=0.012) (71). In solid tumors, other research found that elevated baseline lymphocyte levels were linked to irAEs (47). In patients with melanoma who experienced severe irAEs, peripheral blood samples were evaluated early during treatment, and 11 elevated cytokines were recruited in the validation group for the predictive model (154).

We also evaluated the relationship between the initial steroid dose and OS (**Supplement Figures 1**, **2**). Unfortunately, no significant difference in OS was found among low-dose, intermediate-dose, and high-dose steroid groups. Some reasons might be that the sample size was small and the precise data about steroid doses were limited, so the optimal steroid dose for OS was not determined. Therefore, extensive multicenter studies, which have detailed management of steroid therapy, should be conducted in the future.

## Post-CIP Evolution and Typical Sequela

The evolution of post-CIP patients is largely dependent on their CIP status. Patients with moderate or well-controlled CIP would have various subsequent treatment options including only supportive care, cytotoxic chemotherapy alone and ICIs rechallenge, based on the primary tumor response, irAEs evaluation, and patients’ willingness (155). Yamagata et al. (156) conducted a retrospective analysis concerning the NSCLC patients with CIP and reported the cancer therapy after CIP. They found that 34.6% of CIP patients decided to treat with cytotoxic chemotherapy, and 30.8% of CIP patients chose the best supportive care after CIP. The rechallenge of ICIs only applied on 3% of CIP patients. Actually, if the patients get complete or partial remission (CR or PR), the therapeutic strategies without ICIs could be considered for continued use (157). However, the options of rechallenge should be deliberated in the context of personalized consideration and multidisciplinary evaluation.

Patients with neurologic, cardiac, or any grade 4 irAEs are not recommended to continue or rechallenge ICIs (158). The evaluation of ICIs rechallenge mainly depends on risk-reward ratio (158). At present, there is no acknowledged guidance for re-challenging ICIs. Whether patients should resume ICI monotherapy after receiving doublet ICI therapy is still being investigated. A recent study recruited 80 patients with irAEs on doublet ICI therapy who subsequently reinstated ICIs as monotherapy, and the results indicated that the incidence of CIP (33%) was significantly higher than ophthalmic or gastrointestinal immune-related toxicity (159). However, in most instances, the ICI utilized for re-initiation in NSCLC could be the same ICIs used before, another PD-1/PD-L1 inhibitors, or the switching from PD-1 to PD-L1 inhibitors or the converse (160–164). Kitagawa et al. (157) included prior reports about ICIs rechallenge in NSCLC and analyzed its efficacy and safety. The results showed the generally lower overall response rate (ORR), disease control rate (DCR), and the median PFS presented in patients received the second ICI than in those received the first ICI among these studies. The greatest DCR (58.8%) and longest median PFS (4.0 months) during the second ICI treatment were showed in the 17 patients Kitagawa et al. (157) included. All these 17 patients switched the ICIs type when ICI rechallenge, of which 58.8% obtained PR or stable disease (SD) after switching ICIs administration. However, the efficacy of ICIs rechallenge is still controversial (165–167). Between two ICIs administration, shorter interval may exert better effects on outcome. Besides, the potential predictive factors of ICIs rechallenge outcome include early irAEs development, irAE therapy intensity, CIP phenotype, PD-1 inhibitors, and age more than 65 (155). As for the safety, the second attempt of ICIs could cause same irAEs or moderate new irAEs. Naidoo et al. (168) reported that 3 of 12 patients who reinstated ICI therapy developed CIP recurrence (initial CIP grade of 1 or 2), and 38 of 68 patients developed irAEs after re-treatment. Once patients experience recurrent CIP, the discontinued ICIs in time and the monotherapy of same steroid administrated before is universally acknowledged (19), while with a slower dose tapering and longer course (142).

Notably, there might exist durable anti-tumor activity after discontinuing ICIs therapy (44, 169, 170). This continuous treatment tendency could hold on until intolerable irAEs appearance, tumor progression or no more than 2 years. The correlation of tumor response and toxicity enhances the complexity of ICIs therapy and requires to be demonstrated further. Gauci et al. (170) found that the favorable predictive factors for prolonged response after stopping ICIs therapy included CR patients before discontinuation, with 13% increase of keeping disease stability compared to PR patients.

There are few reports about the sequela of CIP. The typical sequela might be the sustaining pulmonary interstitial fibrosis and poor pulmonary function caused by severe CIP (171, 172). Nintedanib, as an angiokinase blocker, has been reported to play significant role in progressive fibrosing interstitial lung disease, contributing to slow down the decline rate of forced vital capacity (FVC) (173) and further potentially strengthen the prevention of CIP (174).

## Future Directions for CIP

Although quite a few researchers have intensively studied the characteristics of CIP in NSCLC, the studies with regard to the diagnosis, treatment and risk stratification require more exploration (175). First, timely and accurate diagnosis of CIP is necessary. The current biomarkers are based on the mechanism of irAEs. Among the various biomarkers, Isono et al. (176) recently found idiopathic interstitial pneumonias became the only risk factor of CIP in the multivariate Cox regression model. Therefore, the ability of these biomarkers to predict CIP should be investigated deeply.

Second, the management of CIP remains inconclusive. The optimal drug regimen of corticosteroid (taper and continuous time) for CIP and ICIs (onset) for post-CIP need more clinical studies with large sample size to evaluate. Currently, the corresponding two clinical protocols, NCT04036721 and NCT04169503, are ongoing and expected to present profound results.

Third, risk stratification for CIP contributes to precise treatment. CIP presents with different incidence and death rates in different histological types of NSCLC, which may be ascribed to the intrinsic features of tumor histological subtypes (19, 24). Thus, we need more research about the clinical, radiological, histological, and biological characteristics of CIP to determine whether specific subsets of patients should be treated prophylactically.

## Statistics Analysis

We conducted the descriptive analyses to delineate the baseline characteristics and the intergroup differences in different CIP outcomes and CIP grade groups. Kruskal-Wallis test and chi-square or Fisher’s exact test were utilized to analyze continuous and categorical variables, respectively. The former variables were presented by means and standard deviations, and the latter variables were expressed as counts and proportions. The overall and CIP survival rate were estimated by Kaplan-Meier method with a log-rank test. The statistical software packages R and EmpowerStats (X&Y Solutions Inc., Boston, MA, USA) were utilized to conduct all the statistical analyses. Two-sided P-values less than 0.05 were considered to identify statistical significance.

## Author Contributions

QZ and LT searched the literature and wrote the manuscript. YZ helped to collect literature and participated in discussions. LT and WH performed the statistics analysis. WL examined and verified the study. All authors contributed to the article and approved the submitted version.

## Conflict of Interest

The authors declare that the research was conducted in the absence of any commercial or financial relationships that could be construed as a potential conflict of interest.

## Supplementary Material

Supplementary Figure 1 Relationship between initial corticosteroids dose and checkpoint inhibitor pneumonitis outcome. Kaplan-Meier curves by initial corticosteroids dose (mg/d) **(A)**, by initial corticosteroids dose (mg/kg/d) **(B)**.

Supplementary Figure 2 Relationship between initial corticosteroids dose and overall survival. Kaplan-Meier curves by initial corticosteroids dose (mg/d) **(A)**, by initial corticosteroids dose (mg/kg/d) **(B)**.
