# Supplementary figures and images for: Immune Checkpoint Inhibitor-Associated Pneumonitis in Non-Small Cell Lung Cancer: Current Understanding in Characteristics, Diagnosis, and Management
Source: Front Immunol. 2021 May 28;12:663986. doi: 10.3389/fimmu.2021.663986 (PMC8195248; doi:10.3389/fimmu.2021.663986)

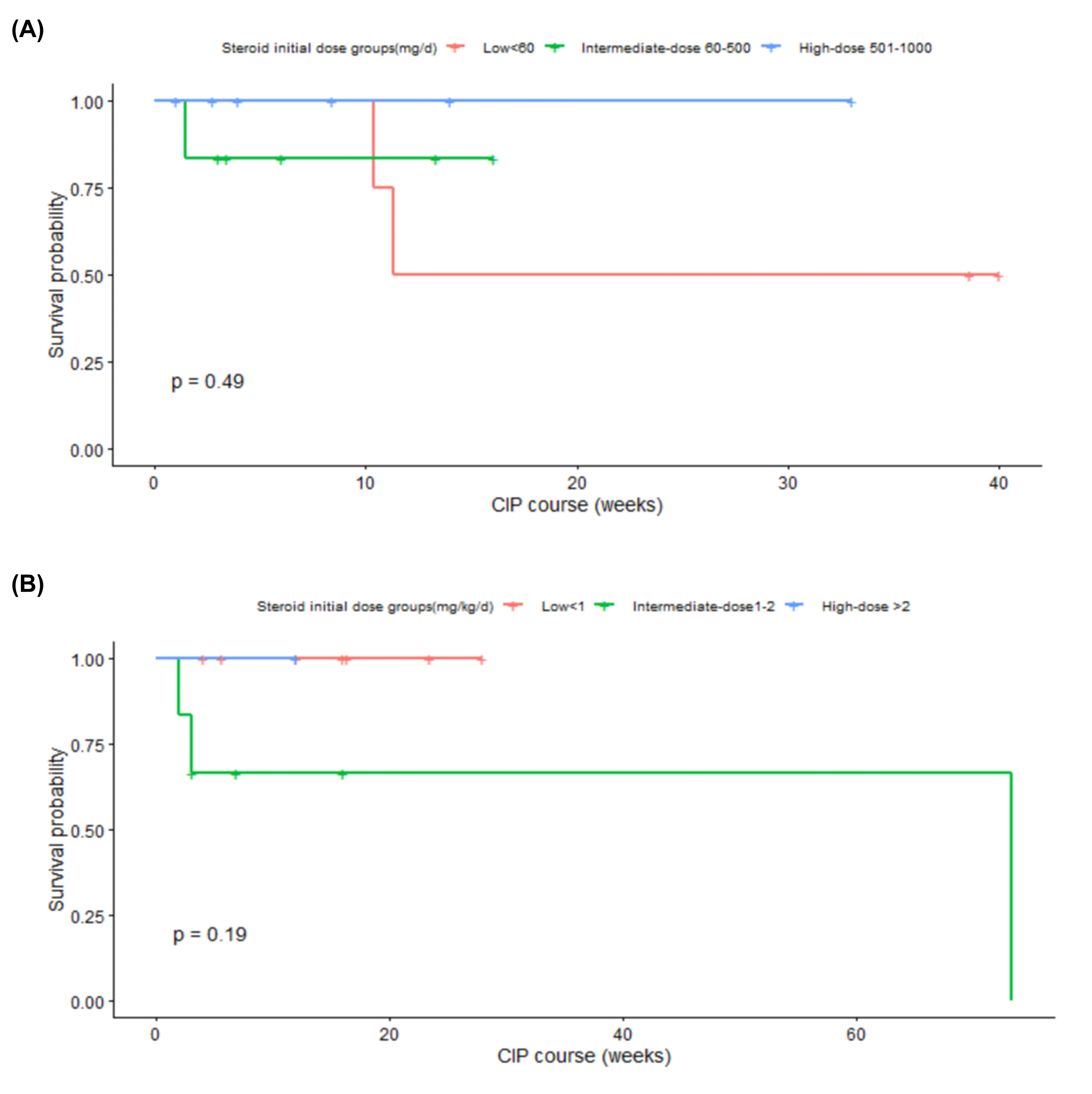

Supplement: Supplementary Figure 1 — Relationship between initial corticosteroids dose and checkpoint inhibitor pneumonitis outcome. Kaplan-Meier curves by initial corticosteroids dose (mg/d) (A), by initial corticosteroids dose (mg/kg/d) (B). [file Image_1.jpeg]

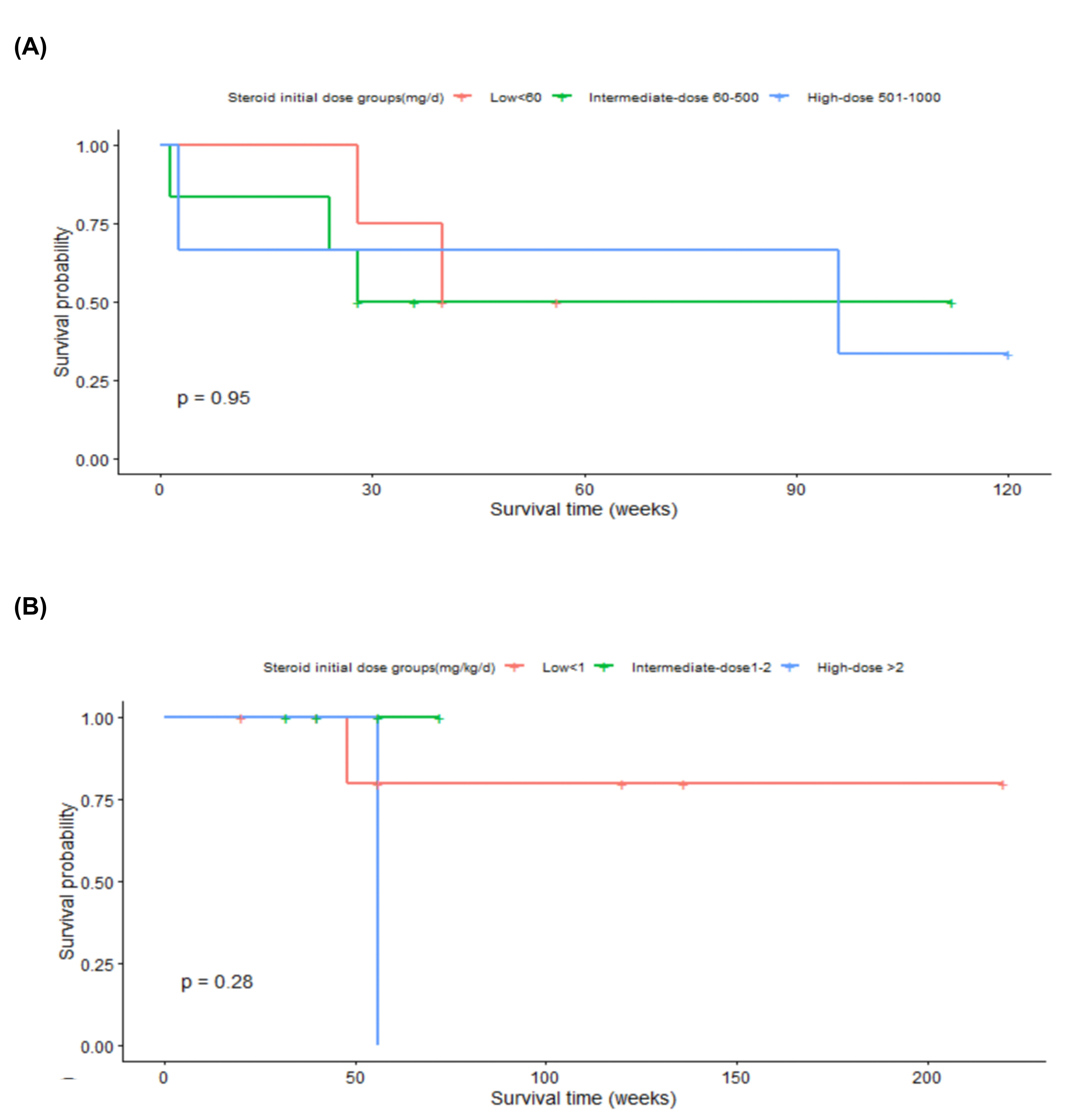

Supplement: Supplementary Figure 2 — Relationship between initial corticosteroids dose and overall survival. Kaplan-Meier curves by initial corticosteroids dose (mg/d) (A), by initial corticosteroids dose (mg/kg/d) (B). [file Image_2.jpeg]
