# Supplementary material for: Immune Checkpoint Inhibitor-Associated Pneumonitis in Non-Small Cell Lung Cancer: Current Understanding in Characteristics, Diagnosis, and Management
Source: Front Immunol. 2021 May 28;12:663986. doi: 10.3389/fimmu.2021.663986 (PMC8195248; doi:10.3389/fimmu.2021.663986)
Supplement: Supplementary file 3 [file DataSheet_1.docx]

**Supplement Table 1**. Symptoms of the NSCLC cases with CIP we included

| **Symptoms** | **N (%)** | **Symptoms** | **N (%)** |
| --- | --- | --- | --- |
| **Dyspnea** |  | **Bloody sputum** |  |
| No | 16 (36.36%) | No | 43 (97.73%) |
| Yes | 28 (63.64%) | Yes | 1 (2.27%) |
| **Cough** |  | **Crackles** |  |
| No | 28 (63.64%) | No | 43 (97.73%) |
| Yes | 16 (36.36%) | Yes | 1 (2.27%) |
| **Fever** |  | **Reduced intake** |  |
| No | 33 (75.00%) | No | 43 (97.73%) |
| Yes | 11 (25.00%) | Yes | 1 (2.27%) |
| **Shortness of breath** |  | **Hypotension** |  |
| No | 39 (88.64%) | No | 43 (97.73%) |
| Yes | 5 (11.36%) | Yes | 1 (2.27%) |
| **Fatigue** |  | **Tachycardia** |  |
| No | 40 (90.91%) | No | 43 (97.73%) |
| Yes | 4 (9.09%) | Yes | 1 (2.27%) |
| **Nausea** |  | **Malaise** |  |
| No | 41 (93.18%) | No | 43 (97.73%) |
| Yes | 3 (6.82%) | Yes | 1 (2.27%) |
| **Diarrhoea** |  | **Palpitation** |  |
| No | 43 (97.73%) | No | 43 (97.73%) |
| Yes | 1 (2.27%) | Yes | 1 (2.27%) |
| **Chest discomfort** |  | **Weight loss** |  |
| No | 42(95.45%) | No | 43 (97.73%) |
| Yes | 2 (4.55%) | Yes | 1 (2.27%) |

**Supplement Table 2**. Different grade definitions of CIP based on ASCO and ESMO

| **ASCO** | | **ESMO** | |
| --- | --- | --- | --- |
| **Grade 1** | asymptomatic aspect | **Grade 1** | radiographic changes only |
| **Grade 2** | symptomatic aspect as a limiting instrumental ADL and indicated medical intervention | **Grade 2** | mid/moderate new symptom occurrence (dyspnoea, cough and chest pain) |
| **Grade 3** | severe symptoms limiting self-care ADL and indicated oxygen | **Grade 3 and Grade 4** | severe new symptoms (new or worsening hypoxia, threat to life and breathing difficulties) |
| **Grade 4** | life-threatening respiratory compromise and indicated urgent intervention |  |  |

ASCO: American Society of Clinical Oncology

ESMO: European Society for Medical Oncology

ADL: Activities of daily living
